# How important is subjective well-being for patients? A qualitative interview study of people with psoriasis

**DOI:** 10.1007/s11136-022-03189-w

**Published:** 2022-08-10

**Authors:** Antonia-Luise Newi, Athanasios Tsianakas, Sophia von Martial, Rachel Sommer, Christine Blome

**Affiliations:** 1 Institute for Health Services Research in Dermatology and Nursing (IVDP), University Medical Center Hamburg-Eppendorf (UKE), Hamburg, Germany; 2 Department of Dermatology and Allergology, Specialized Hospital Bad Bentheim, Bad Bentheim, Germany

**Keywords:** Treatment benefit, Psoriasis, Happiness, Subjective well-being

## Abstract

**Purpose:**

This qualitative study aimed to investigate the importance of subjective well-being (SWB) as an outcome of psoriasis treatment from patient’s perspective. We focused on the affective component of SWB as assessed with the Daily Experience Sampling Questionnaire (DESQ), a validated daily diary.

**Methods:**

Semi-structured qualitative telephone interviews were conducted with in-patients of a dermatological rehabilitation clinic, after participants had completed the DESQ for up to seven days to get familiar with the concept of SWB. Patients were asked to reflect on the importance of SWB as treatment goal and on its relative importance as compared with other treatment outcomes. We also addressed whether SWB could be an indirect measure of benefit in that it reflects other important outcomes. Transcripts were analyzed using content analysis.

**Results:**

Eleven patients participated (24–63 years, mean 53 years, 8 male, 3 female). Participants uniformly confirmed that changes in SWB reflected treatment benefit. All but one considered SWB to be a central aspect of treatment benefit—either as the most important treatment goal or as an indirect benefit indicator. In particular, participants described positive associations of SWB with other outcomes, such as symptoms. They reported that both the disease and the medical treatment had an impact on their SWB, which was reflected in the DESQ.

**Conclusion:**

Our findings suggest that SWB is a relevant indicator of treatment benefit for patients with psoriasis. Therefore, SWB measures, such as the DESQ, could be used to operationalize patient-relevant benefit of psoriasis treatment, complementing outcome measures currently used.

**Supplementary Information:**

The online version contains supplementary material available at 10.1007/s11136-022-03189-w.

## Introduction

Psoriasis is an incurable, chronic autoimmune skin disease characterized by inflammation, skin lesions, and a variety of associated comorbidities [1, 2]. In Germany, between 2.5 and 2.8% of the population is affected by psoriasis [3, 4]. The disease mostly occurs in adults with rising incidence up to the age of 39 years [5]. Typical symptoms include scaling, rash, severe itching, plaque formation (sharply defined redness of the skin), skin pain, and bleeding. In addition to physical symptoms, psoriasis places similar psychosocial burden on patients as cardiovascular diseases or cancer [6–8].

Research suggests that impaired well-being caused by psoriasis is less related to objective measures of disease severity but rather to patients' subjective perception of symptoms [9–11]. Several studies show that the symptoms have strong negative impact on patients' cognitions and emotions about themselves and their lives [12–14]. This might be a reason why evaluations of a dermatologic intervention differ between doctors and patients [15]. Consequently, consideration of patient experience is essential in assessing the effectiveness of psoriasis treatment. For this, quality of life (QoL) as defined by WHO and health-related quality of life (HRQoL) are well-established concepts [16–18]. As a multidimensional concept, the broader term QoL incorporates environmental, social, material, and other aspects of life, while HRQoL focuses on the effects of illness and treatment on an individual’s perceived health [19, 20]. However, it has been questioned how much these indicators really capture the full life impact of a long-term condition such as psoriasis [21]. As a more holistic indicator of patient benefit, subjective well-being (SWB) has been proposed [22, 23].

SWB refers exclusively to the subjective perspective by simply asking how people feel or think about their lives. Affective SWB—the extent of positive and negative feelings on a daily basis—or cognitive SWB—the evaluation of overall satisfaction with life—thus takes into account that circumstances which are functional for a good life may vary [24–26]. In the context of patient-centered care, the degree to which a medical intervention supports patients pursuing life goals can be defined as patient benefit [27]. Diener et al. (1998) argue that positive affective SWB “results from people having a feeling of mastery and making progress toward their goals” [28]. Conclusively, affective SWB increases, when patient-relevant goals are fulfilled [29]. For this reason and because affective SWB has a particularly high value for humans [28], this study focused on affective SWB as a potential indicator of patient-relevant benefit.

Measuring affective SWB in addition to HRQoL may provide valuable insights of the subjective full life impact of a disease. Schuster and colleagues showed that both affective and cognitive SWB are reduced in psoriatic patients, while lower levels of affective well-being indicate a higher risk for depression [30, 31]. Reimus and colleagues revealed that SWB and dermatologists assessment of illness severity are not related [10]. Both studies concluded that assessment of SWB can provide a better understanding of the mental burden of psoriasis. However, information on the importance of affective SWB for psoriatic patients and the relationship between affective SWB and other treatment outcomes is limited. For the first time, this study gives some insight into patients’ perspective on measuring treatment benefit in relation to affective SWB.

Knowledge of the relevance of SWB as indicator of treatment benefit can enhance our understanding of measuring what is truly relevant to patients, may improve the psychological management of psoriasis, and can strengthen the clinical view on less visible aspects of well-being.

The aim of this study was to research the relevance of affective SWB as indicator of treatment benefit in people with psoriasis using the “Daily Experience Sampling Questionnaire” (DESQ), a validated daily diary assessing positive and negative emotion [32]. Specifically, we addressed the following questions:

1. How relevant is affective SWB as a benefit parameter of psoriasis treatment from the patients’ perspective?
2. How do patients describe the relationship between affective SWB and other outcomes relevant to them?
3. From the patients’ perspective, how important is affective SWB compared to other treatment outcomes?

## Methods

This was a qualitative study using semi-structured one-on-one telephone interviews.

Patients with psoriasis were recruited at an in-patient rehabilitation clinic in Germany. The sample should be heterogeneous with respect to gender, age, and disease severity. Inclusion criteria were diagnosed psoriasis, age of majority (≥ 18 years), sufficient cognitive and linguistic ability, and written informed consent. Participants did not receive financial remuneration. Dermatologists at the rehabilitation clinic in Bad Bentheim invited in-care patients to participate in the study, after which A.-L. N. contacted these patients to schedule the interview.

### Sample size

We estimated a sample size of 8 to 16 interviews to reach code saturation. Code saturation is achieved when all relevant themes in the data have been identified, in contrast to meaning saturation, which implies that no more interviews are needed for a complete understanding of the themes [33–35]. The estimation was based on study characteristics known to affect saturation of the data, such as homogeneity of study population and degree of interview structure [34, 36, 37]. We assumed that our sample would be fairly homogeneous, as all patients received therapy at the same clinic, and that the data would have limited complexity due to the semi-structured interview guideline. Both contribute to a densified data quality and stability of the codebook [34].

### Materials

We used hypothetical scenarios, visualization tasks, and prompts (see Online Appendix 1). Tasks like these had provided important additional information in previous qualitative studies in psoriasis, especially regarding emotional topics [38, 39]. After the interview guideline had been pilot tested in face-to-face interviews by C. B., the pandemic situation in winter 2020/2021 required a shift to telephone interviews with minor adjustments to the guideline. Before the interview, participants completed the DESQ during their clinic stay over a period of up to seven days.

### Interviews

The telephone interviews were conducted by A.-L. N., who had experience in conducting qualitative interviews and was supervised by C. B.

At the beginning, we requested one word what participants thought the DESQ measures and used this term throughout the interview. After an open question on the meaning of the term in relation to psoriasis, we asked whether psoriasis influenced patient’s actual responses in the DESQ. If the disease was currently not active, we were also interested in the impact they thought an active disease state would have had on their responses. Then, we presented three scenarios which described different trends of DESQ responses over a period of several weeks after onset of a new treatment: constant, worsening, or improving (see scenario 1 to 3 in Online Appendix 1). Patients should answer whether the response patterns would indicate anything about treatment success.

Next, we asked how patients can tell whether their treatment has been successful; if they did not mention SWB here, but other aspects of treatment such as less itching, we also asked for the importance of SWB in describing success. Further, we addressed whether affective SWB was suitable as an indirect measure of overall treatment benefit in that it may reflect other outcomes important to them.

For the investigation of the relative importance of SWB as a treatment goal, we used one visualization task and two scenarios (see scenario 4 and 5 in Online Appendix 1). The visualization was introduced by the imagination of cards of different size lying in front of the interviewee. The largest card represented the most important of the outcomes mentioned by the patient whereas the smallest card represented the least important. Participants should assign their stated treatment goals to the cards according to their importance. In addition, patients should indicate possible connections between the outcomes. In the fourth scenario, we launched the image of two physicians who expressed different opinions on the importance of SWB and let participants choose whom they agreed with. Finally, in scenario five, participants had to decide between hypothetical therapy options, each of which was only known to improve one of the outcomes named by the patient as relevant, without having any serious side effects.

At the end, the interviewer documented demographic and treatment characteristics, as well as self-perceived psoriasis severity. A.-L. N. ensured no one but her was present during the telephone interviews. Data collection took place between November 2020 and January 2021. All interviews were audio-recorded and lasted about 45 min.

### Data analysis

A.-L. N. transcribed recordings verbatim and imported the data to NVivo 12 (QSR International, Doncaster, Australia). We analyzed the data primarily inductively using an adapted approach to content analysis with the aim of developing a category system relevant to our research questions [40, 41]. In a first step, we read the transcripts line by line and paraphrased relevant text units. At this level, each code name was formed by paraphrasing, thus on a purely descriptive level of the essential content of this passage. In a second step, we reduced the number of paraphrases within each interview by merging the ones with the same meaning in one code. In an iterative process, recurring themes were grouped into codes across the individual transcripts. For this purpose, thematically similar answers were combined in a code named after the theme of the interview questions (which presents a deductive element in the analysis). Newly addressed topics related to the importance of SWB were coded additionally. In a final step, all codes were assigned to the three research questions, so that three upper categories were formed (see Table 2 in Online Appendix 2). To ensure consistency in the coding process, results were regularly discussed with C. B.

## Results

Of 13 patients recruited, two did not participate in the study because they were not available at the provided telephone number. In total, 11 participants completed the DESQ for five to seven days (mean 6.4 days). Sample characteristics are presented in Table 1.

**Table 1:** Participant characteristics

	*n*	%
*Education*		
Upper secondary school certificate	6	54.5
Lower secondary school certificate	3	27.3
A-levels	1	9.1
Not indicated	1	9.1
*Psoriasis types*		
Psoriasis not further specified	6	54.5
Psoriasis and psoriasis arthritis	2	18.2
Psoriasis vulgaris	3	27.3
*Psoriasis severity*		
Mild	2	18.2
Moderate	6	54.5
Severe	3	27.3
*Gender*		
Male	8	72.7
Female	3	27.3

	Mean	Standard deviation	Range
Age (years)	52.9	10.9	63–24
Years of psoriasis diagnosis	21.2	12.3	3–34

In the following, we present the identified themes with examples of patients’ responses. For an overview, please see (see Online Appendix 2).

### Subjective well-being as treatment outcome

#### Terms for SWB

Asked about the concept measured by the DESQ, four participants chose the term “well-being”, two “condition” and one each “feel-good-factor”, “today’s emotion”, “feel-good behavior”, and “feeling good”. The eleventh person did not name a term, saying he did not know what to respond. This participant also described the questionnaire as “irritating” and “childish” (male, 62 years).

#### Influence of disease and treatment on SWB

Ten out of the eleven patients reported that the disease had an impact on their SWB or, more exactly, on the concept they named as being measured by the DESQ. This applied to both patients with active psoriasis at the time of completing the questionnaire and patients with an inactive disease state due to successful therapy during their in-patient stay. The latter assumed that an active disease state would have negatively affected their DESQ responses. In the eleventh person, illness worsened during the DESQ completion period, but she stated that this did not affect her answers, because everything possible had been done to cure her illness during rehabilitation:

No no. [When asked whether the illness had an influence on DESQ responses] Uh, in regard to the illness itself is concerned, everything is being tried indeed, I would say. (Female, 52 years)

To check whether DESQ responses reflect treatment benefit, we analyzed the answers to the first three scenarios: All participants confirmed that the different hypothetical DESQ response trends reflected treatment success, linking SWB improvement to higher benefit and SWB deterioration to lower success. However, results were less clear for the constant responses scenario. Some participants assigned these to “resignation” or stated they would just give the treatment “a little time.” One patient noted that previous experience played an important role in what constant responses indicate:

Then [if SWB response remained constant] I would also blame it on the treatment. (...) Unless I knew from experience it could be much worse (...). It's always a matter of the perspective from which you look at it – isn’t it? If you have this attitude or already made very, very bitter experiences, then you're glad if you never reach this bitter point again. (Male, 47 years)

In addition to the effects of treatment on SWB, patients mentioned other psoriasis-related factors that modify SWB, and thus treatment success, in the context of their daily lives or rehabilitation. They described these factors to either change disease status and consequently SWB or affect SWB but not necessarily the symptoms. For example, the availability and feasibility of effective therapies (*n* = 4), disease relapses (*n* = 3), stress (*n* = 3), personal calmness (male, 47 years; *n* = 1), and seasons (male, 58 years; *n* = 1) were found to change disease status and SWB, whereas expectations of a new therapy (*n* = 4), interaction with peers during in-patient stay (*n* = 4), physical activity (*n* = 4), patient education (*n* = 4), or distraction from the disease (*n* = 1) affect SWB but not necessarily the symptoms.

### Relevance of SWB as an indicator of treatment benefit

When asked what outcomes are crucial for therapy success, and, if not spontaneously named, for the role of SWB in describing success, eight participants considered affective SWB as important.

Yes, actually a relatively large one [role of SWB for treatment benefit], let’s put it that way. Because if you don’t feel comfortable with yourself, how are you going to face other people or look at yourself in the mirror if you’re not happy with yourself? (Male, 58 years)

Three participants did not spontaneously mention the importance of SWB as an outcome, but described SWB to have a different role, for example, as a cause of therapy success. However, no one said that SWB was not important or worthwhile.

### Relationship between SWB and other treatment outcomes

#### Relevant outcomes of psoriasis therapy

Besides SWB, patients specified only symptom-related outcomes as relevant for therapy success. In descending frequency, these outcomes included skin appearance (*n* = 10) including dandruff and inflammation, itching (*n* = 4), pain (*n* = 4), SWB (*n* = 4), inflammation in joints and other organs (*n* = 2), and hair loss (*n* = 1). Some participants mentioned SWB as an indicator of successful treatment in itself, stating, for example, that a characteristic of successful treatment was “simply the (…) own well-being” (male, 58 years). Others combined SWB and skin appearance: “When I feel good in my skin.” (Female, 63 years).

#### Relation between SWB and relevant treatment outcomes

When asked about relationships between the outcomes named as important by the respective patient, ten persons reported that a deterioration or improvement of symptom-related outcomes had a corresponding impact on their SWB. Some described direct effects of symptoms such as skin appearance, itching, or pain on their SWB in that any change would be depicted in their DESQ responses:

And the skin has also improved. So, I also rate the smileys a bit higher. (Male, 62 years)

So I think the itching, that is what has an extreme impact on the daily schedule [patient’s term for DESQ questionnaire]. (Male, 50 years)

Most participants (*n* = 9) also described indirect influences of symptoms on SWB, mainly by the extent and visibility of skin appearance. Many reported negative reactions to visibly affected body parts, severe scaling, or hair loss (*n* = 5):

And even if your own work colleagues then say, ‘You, I’ve already thought about whether I may even still shake your hand at all?’ (Male, 58 years)

In general, these patients reported that skin symptoms in less visible areas had less impact on their SWB:

Yes, it [the psoriasis] was on body areas where you did not see it. And then, to be quite honest, it didn’t bother me so much. (Female, 52 years)

As a possible consequence of negative reactions from others and high visibility of affected skin areas, two interviewees also mentioned social isolation with regard to a reduced SWB:

You feel really MISERABLE. So, you really only go out in the dark (…) if you have the entire back of your head, everything damaged, open (...) and from your neck to your knees in one (...) practically red all the way through and, uh, everything is open, uh, it does look bad. You have no real life there. (Male, 55 years)

Finally, nine participants considered SWB to be an indicator of disease status, as improved SWB reflects improvements in other outcomes as well.

### Relative importance of SWB

#### Relative importance of relevant treatment outcomes

Concerning the importance of SWB compared to further outcomes named by patients, interviewees assigned SWB to the largest card (*n* = 3), the medium (*n* = 2), or the smallest card (*n* = 4). One person did not assign any importance to SWB, while another assigned equal importance to the outcomes pain, skin appearance, and SWB. Generally, we found that the most important therapy outcomes were the same as those most frequently mentioned as factors that influence SWB.

In the drug scenario (scenario 5), the majority of participants chose a medication with a symptom-related effect (*n* = 9), while three chose a drug that improved SWB. For many respondents, the answer to this scenario was congruent with their response to the visualization task. However, responses deviated in four of the 11 patients.

Taken together, we found that five people indicated SWB as the most important outcome in at least one of the two tasks (visualization and/or scenario 5). One person indicated it as the second important outcome, and four persons as the least important outcome. These four persons nevertheless described SWB to be a major therapeutic goal. For one person, SWB did not seem to be a significant aspect of treatment benefit, as he did not mention it in either of the two tasks.

#### Excluded scenario

In this scenario (scenario 4, describing the opinion of two physicians), very few responses were informative regarding the relative importance of SWB. Instead, most interviewees gave arguments that were not related to SWB. For example, some emphasized that as a matter of principle, they would not take a drug in order to improve their SWB. Others expressed personal attitudes that affected their decision such as a general skepticism about drugs or their general trust in their physicians. We therefore considered this scenario to be little informative for our research questions.

## Discussion

In this study, we asked people with psoriasis to reflect on the importance of SWB as a treatment outcome. All but one of the 11 participants considered affective SWB to be an important therapy goal, either in itself or as an indirect measure of other outcomes, such as symptoms. The latter became particularly evident in participants’ explanations of associations between the treatment outcomes: Almost all described indirect and direct relations between symptom-related outcomes and SWB. Participants unanimously considered the DESQ as indicator of treatment benefit affected by both treatment- and not-treatment-related changes. Half of the participants saw SWB as a prerequisite for potential treatment success.

### What this study adds

To our knowledge, the present study is the first qualitative investigation of patients' relevance on affective SWB in dermatology. Previous, quantitative studies found that SWB plays an important role in psoriasis, with SWB being lower than in healthy controls [30] and associations between emotions and skin lesions [12]. In the present study, patients described SWB to be associated with changes in symptoms, especially skin appearance. Our study extends the previous findings by gaining in-depth patients’ perspectives when making judgements about the effectiveness of their therapy. We show that SWB is of high value in comparison with symptom-related outcomes for people with psoriasis. This finding is consistent with a qualitative study by Ersser et al. (2002) which used in-depth structured interviews and found that patients focus on subjective perception of symptoms when judging the effectiveness of psoriasis treatments [42].

In our study, patients pointed to changes in their SWB and thus their treatment success that were independent of merely drug treatment, for example, through stress or interaction with peers during in-patient stay. Patients mentioned that these factors improved their SWB during rehabilitation, but they described them as a greater obstacle to higher SWB and treatment success in their daily living environment. These results are in line with previous studies in psoriasis that not only underline the need of a holistic approach to psychosocial care in psoriasis but also the need for a more holistic assessment of patient benefit [39, 43, 44]. Here, the DESQ could be used as a patient-reported outcome in addition to measures of HRQoL or as one main indicator which many patients see as reflecting the overall benefit of treatment.

People with chronic illnesses seem to strive for a high well-being without necessarily distinguishing between the aspects of their lives that are affected by health and those that are not. From the patient’s point of view, the narrow focus on HRQoL may therefore seem artificial while looking at SWB more closely better matches their personal goals and their expectations of treatment. For these reasons, researchers are increasingly advocating the use of benefit parameters more global than HRQoL—for example, SWB [45, 46]. Moreover, patient well-being correlates positively with physician well-being, and the latter in turn may have positive implications for quality of care [47, 48].

#### Strength and limitations

This study is reported according to the consolidated criteria for reporting qualitative research (COREQ) [49]. As a strength, we discussed SWB based on how it was measured, asking patients to complete the DESQ questionnaire in advance and used the patients’ own term for SWB throughout the interviews. Patients filled in the questionnaire for an average of 6.4 days, which may have deepened their understanding of the concept.

One challenge in investigating SWB as therapy outcome was that people have probably never dealt with the relevance of this outcome and therefore cannot readily assess it. Therefore, we decided to use various scenarios and tasks to support patients’ individual reflection on their main therapeutic goals in concrete, though hypothetical, choice situations. We believe that our results would not have been as robust without these specific tasks in the interview guide. In addition, our results show that the open questions after each scenario provided valuable information on the participants’ rationales and thoughts, which underlines the need for a qualitative study design.

The finding that half of the respondents did not name SWB but a symptom-related outcome as most important for their therapy success may at first seem to contradict its importance. However, in the following conversations, it became apparent that SWB was actually a major treatment goal for some of these patients, too, even if they had named it least important. One reason might be that symptom-related outcomes are directly observable and more tangible than SWB. They are also much discussed during in-patient rehabilitation and, thus, be more mentally present than SWB.

However, our results may be biased in favor of the importance of SWB, as this concept was already introduced at the beginning of the interviews asking participants what the DESQ measures, only after which we asked for relevant outcomes of psoriasis therapy. SWB may thus have come to mind more easily when we asked for participants’ therapy goals. Another limitation is the limited generalizability of the results to patients with mild psoriasis, as we only interviewed patients with a history of severe psoriasis that indicated in-patient treatment (nine out of 11 reported moderate to severe psoriasis).

Further research is needed to bring the DESQ to application in science and practice. While this study has supported the relevance of the construct measured with this instrument, and previous studies indicated good validity, reliability, and feasibility of the DESQ [32], its responsiveness to change is yet to be investigated. Furthermore, future research should examine the relevance of affective SWB by clinicians.

## Supplementary Information

Below is the link to the electronic supplementary material.

Supplementary file1 (DOCX 35 kb)

## Data Availability

Available from authors upon reasonable request.
