# Supplementary material for: How important is subjective well-being for patients? A qualitative interview study of people with psoriasis
Source: Qual Life Res. 2022 Aug 10;31(12):3355–63. doi: 10.1007/s11136-022-03189-w (PMC9587968; doi:10.1007/s11136-022-03189-w)
Supplement: Supplementary file 1 — Supplementary file1 (DOCX 35 kb) [file 11136_2022_3189_MOESM1_ESM.docx]

**Appendix**

**How important is subjective well-being for patients? A qualitative interview study of people with psoriasis**

Antonia-Luise Newi, PD MD Athanasios Tsianakas, MD Sophia von Martial, PHD Rachel Sommer, PHD Christine Blome

Corresponding author:

Antonia-Luise Newi

Institute for Health Services Research in Dermatology and Nursing (IVDP)

University Medical Center Hamburg-Eppendorf (UKE)

E-mail: a.newi@uke.de

**Appendix 1**

**Interview guide DESQ-Relevance**

*Italics* = content is given analogously; **“bold in inverted commas”** = verbatim presentation

|__|__|__|__|

**ID**

**Today’s date:** |__|__|**.**|__|__|**.**|__|__|__|__|

**material**: interview guide;
blank copy of the DESQ;
demographic questionnaire;
recording equipment;
note paper and pen for interviewer

**Introduction:**

- Welcome and introduction (interviewer)
- Explanation of the aim of the study:
  - We want to better understand how important different aspects to people with psoriasis in relation to their psoriasis condition are.
  - The results will be used to find out what and how should be measured in clinical trials to best describe the benefits for patients
- Procedure of the interview, expected duration, again reference to pseudonymization (= code instead of name to exclude identification of the study participants) and use of the audio recording (access protection; transcript without personal information; deletion date after 3 yrs.).
- Voluntary participation and the possibility to stop during the interview
- Point out that there are no right or wrong answers, but that we are interested in the subjective view of the respondent
- Indication that we are happy to repeat all questions
- Preliminary information: position as a neutral interviewer in order to avoid directionality in answers; possibility to discuss open questions at the end of the interview
- Ask if questionnaire is in front of the participant

**Main part:**

- *You have been filling out this questionnaire for a few days now (DESQ questionnaire). If you had to describe it in one word, what was asked for there? That is, if you had to find a term for what was measured there, what would it be called?*

Prompts: If no concrete term is named by the respondent: *What do you think the smileys question asks for? Can you describe it in your own words? What do you think is being asked with the seven possible smileys? What would you personally describe as what you were asked every day in this questionnaire or what you answered there?*

- *In the questionnaire you were asked how you feel. Your answers can be related to different things. This interview is about your answers in relation to your psoriasis.*
- **“What does [** **
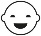
** = term mentioned by respondent in question 1**] mean to you in relation to your psoriasis?”**
- Prompts: *What role does what was asked in the questionnaire play for you personally in relation to your psoriasis?*
- **“Do you think your condition had any influence on your answers?”**

a. If “yes” and not named spontaneously: **“In what way?”**

b. If “no” and not spontaneously stated: **“Why not?”**

c. If condition not currently active: **“If your condition had been active at the time you filled in, what impact would it have had on you?”**

- Scenario 1: **“Suppose your psoriasis is currently very active and you get a new treatment and fill in this questionnaire over several weeks. Over the whole period you answer the questions mostly the same. Does this say anything about the success of your treatment, or would you think that this has rather nothing to do with the treatment?**”
- Scenario 2: **“Imagine you have a new treatment and fill in the questionnaire over several weeks. In the first period you tend to tick the smileys on the left side (for the “How do you feel” question). Towards the end, more often the ones on the right. Does this say anything about the success of your treatment?”**

If not understandable: your answers move more and more from ‘Bad’ to ‘Good’.

- Scenario 3: **“Imagine you have a new treatment and fill in the questionnaire over several weeks. Over the weeks your answers go more and more from the right smileys to those on the left. Does this say anything about the success of your treatment?”**

If not understandable: your answers go more and more in the direction of ‘good’ to ‘bad’.

- **“What do you personally think is crucial to the success of your psoriasis treatment?”**

Prompt: **“How can you tell if your treatment was successful?”**

Prompts: If respondent does not mention any specific aspects: **“When is the treatment of psoriasis successful for you?”** or:

1. _______________________ 2. ________________________ 3._______________________

- **“How important is [
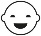
] to you personally in describing the success of your psoriasis treatment?”**

Prompt: **“How important is [
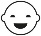
] to you personally in terms of the benefits of your psoriasis treatment?”**

*If [****
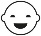
****] is better, does that mean for you that all other aspects, e.g. [*mention here an aspect previously mentioned by the patient*] are also better?*

- **“What else should the treatment improve?”**

Prompt: **“What other aspects besides [
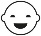
], which is measured in the questionnaire, are important for you personally to say that your treatment is successful?”** and **“Are there any other aspects of what your medical treatment should improve?”**

- Visualization task: *Imagine that you have three cards of different sizes in front of you (large, medium, small). The large card stands for a very important success feature of your treatment, the medium one for important and the small one for a less important success feature. [*Interviewer names the terms*]. Which term would you write on which card?*

If several terms are assigned to the same card size: *Can you rank them [*name corresponding terms*] in terms of importance?*

**“Do you think there is a connection between the individual aspects?”**

If yes: **“Which?”/“In what way?”**

- Scenario 4: **“Imagine you have severe psoriasis. There is a drug that you only know, based on studies, that it improves [
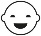
] and that it has no dangerous side effects; that’s all you know.”** *You meet two doctors who have the following opinion about the drug:*

*One doctor says: The drug is certainly good, but you need to know other things to recommend it.*

*The other doctor says: The drug can only be very good if [****
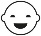
****] is improved.*

**“Which doctor would you go with? Why?”**

- Scenario 5: *Imagine there are [*number*] different medicines. Of the first one you only know that it improves [1. outcome] and has no dangerous side effects; of the second one you only know that it improves [2. outcome] and has no dangerous side effects; and of the third one you only know that it improves [3. outcome] and has no dangerous side effects, etc.* [insert in each case randomly one of the most important aspects mentioned by the patient for the evaluation of success]. *Which of the [*number*] medicines would you choose?*

**Conclusion of the interview:**

- Thank you
- *Do you have any questions or is anything still open for you now?*
- Offer to send the results to the respondent after the evaluation; interest:
  - Yes
  - No
  - Note email address or postal address: ________________________________________
- Farewell

**Appendix 2 (English version)**

*Themes found in the qualitative analysis*

| Theme | Description | Evidence from the data |
| --- | --- | --- |
| 1. Subjective well-being (SWB) as treatment outcome |  |  |
| - 1. Terms for SWB | Patient’s own term for SWB as measured in the Daily Experience Sampling Questionnaire (DESQ) | “well-being” (e.g. male, 58 years)  “condition” (e.g. female, 52 years) |
| - 1. Influence of disease and treatment on SWB | Statements illustrating the influence of disease and treatment on DESQ responses | P: “That’s why I ticked everything between ‘good’ and ‘very good’, because the treatment also worked well relatively quickly.” (male, 60 years) |
| - 1. Relevance of SWB as indicator of treatment benefit | Statements on the importance of SWB – either reported spontaneously or in response to the question about the importance of SWB in describing therapy success | “Yes, actually a relatively large one [role of SWB for treatment benefit], let’s put it that way. Because if you don’t feel good about yourself, how do you want to face other people or look at yourself in the mirror if you’re not happy with yourself?” (male, 58 years) |
| 1. Relationship between SWB and other treatment outcomes |  |  |
| - 1. Relevant outcomes of psoriasis therapy | Treatment outcomes specified by patients when they were asked how in general they can tell whether their treatment has been successful | I: “(…) how do you know that a treatment has been successful for you?”  P: “Yes, that is, as already mentioned many times, just the (...) own well-being.” (male, 58 years) |
| - 1. Relationship between SWB and relevant treatment outcomes | Statements indicating a possible connection between the outcomes; answer to the question whether affective SWB was suitable as an indirect measure of overall treatment benefit in that it may reflect other outcomes important to patients | “Um, so I think the itching, that’s the thing that has an extreme impact on the daily schedule [DESQ questionnaire].” (male, 50 years) |
| 1. Relative importance of SWB |  |  |
| - 1. Relative importance of relevant treatment outcomes | Answers to the visualization task with cards of different size and the drug scenario (scenario 5) | I: “(…) I’ll summarize that again now. We had ‘hair’ on the largest and most important card as a success indicator, then ‘itching’...”  P: “Yes”.  I: “Then ‘skin and dandruff’.”  P: “Mhm” (affirmative)  I: “...then ‘decrease in visible parts of the body’ and then ‘condition’?”  P: “Yes.” (female, 52 years) |
| - 1. Excluded scenario | Answers to scenario 4 that presented the views of two physicians on a drug that is only known to improve SWB | P: “Then [if drug exclusively improves SWB] I would omit it. (...), because I generally keep what I don’t need 100 per cent of the medication out of my body. (...) I don’t trust drugs.” (female, 52 years) |

**Appendix 3 (German version)**

*Identifizierte Themen der qualitativen Analyse*

| Thema | Beschreibung | Belege aus den Daten |
| --- | --- | --- |
| 1. Subjektives Wohlbefinden (SWB) als Erfolgsmerkmal |  |  |
| - 1. Begriffe für SWB | Eigene Bezeichnung des Patienten für SWB, gemessen mit dem „Daily Experience Sampling Questionnaire (DESQ)“ | “Wohlbefinden” (z. B. m., 58 J.)  “Befinden” (z. B. w., 52 J.) |
| - 1. Einfluss der Krankheit und der Behandlung auf SWB | Alle Aussagen, die den Einfluss von Krankheit und Behandlung auf die DESQ-Antworten beschreiben | „Darum habe ich auch alles zwischen „gut“ und „sehr gut“ angekreuzt, weil auch die Behandlung relativ schnell gut anschlägt.“ (m., 60 J.) |
| - 1. Wichtigkeit von SWB als Indikator für den Behandlungsnutzen | Alle Aussagen zur Bedeutung von SWB - entweder spontan oder als Antwort auf die Frage nach der Bedeutung von SWB bei der Beschreibung des Therapieerfolgs | „Ja, eigentlich einen relativ großen, sagen wir es mal so. Weil wenn man sich selbst nicht wohlfühlt, wie will man dann anderen Menschen gegenübertreten oder sich selbst im Spiegel betrachten, wenn man mit sich selbst nicht zufrieden ist?“ (m., 58 J.) |
| 1. Zusammenhang zwischen SWB und anderen Behandlungsergebnissen |  |  |
| - 1. Relevante Erfolgsmerkmale einer Psoriasis-Therapie | Patientenberichtete Behandlungsergebnisse als Antwort auf die Frage, woran sie im Allgemeinen erkennen können, ob ihre Behandlung erfolgreich war | I: „(…) woran erkennen Sie, dass eine Behandlung erfolgreich war für Sie?”  P: „Ja, das ist, wie schon des Öfteren erwähnt, einfach nur das (…) eigene Wohlbefinden.” (m., 58 J.) |
| - 1. Zusammenhang zwischen SWB und relevanten Erfolgsmerkmalen | Aussagen, die auf einen Zusammenhang zwischen den Erfolgsmerkmalen hinweisen; Antworten auf die Frage, ob das affektive SWB als indirektes Maß für den Gesamtnutzen der Behandlung geeignet ist, indem es andere, für die Patienten wichtige Ergebnisse, widerspiegelt | „Also ich denke, der Juckreiz, das ist das, was sich extrem auf den Tagesplan [der Begriff des Patienten für den DESQ Fragebogen] auswirkt.” (m., 50 J.) |
| 1. Relative Wichtigkeit von SWB |  |  |
| - 1. Relative Wichtigkeit relevanter Erfolgsmerkmale | Alle Antworten auf die Visualisierungsaufgabe mit unterschiedlich großen Karten sowie das Szenario 5, bei dem sich die Teilnehmer zwischen hypothetischen Therapieoptionen entscheiden sollten, die jeweils ein relevantes Erfolgsmerkmal verbessern würden | I: “ (…) ich fasse das jetzt noch mal zusammen. (lacht) Wir hatten auf der größten und wichtigsten Karte als Erfolgsmerkmal die Haare, danach den Juckreiz...”  P: „Ja.“  I: „Danach Haut und Schuppen.”  P: “ Hm (bejahend)“  I: „Danach Rückgang an sichtbaren Körperpartien und danach Befinden?”  P: „Ja.” (w., 52 J.) |
| - 1. von der inhaltlichen Analyse ausgeschlossenes Szenario | Alle Antworten auf Szenario 4, in dem die Ansichten von zwei Ärzten zu einem Medikament dargestellt wurden, von dem nur bekannt ist, dass es SWB verbessert | P: „Ja, weil ich ganz generell tunlichst, was ich nicht zu 100 Prozent brauche an Medikamenten eigentlich aus dem Körper lasse (...) Ich vertraue Medikamenten nicht.” (w., 52 J.) |
